# SOST Inhibits Prostate Cancer Invasion

**DOI:** 10.1371/journal.pone.0142058

**Published:** 2015-11-06

**Authors:** Bryan D. Hudson, Nicholas R. Hum, Cynthia B. Thomas, Ayano Kohlgruber, Aimy Sebastian, Nicole M. Collette, Matthew A. Coleman, Blaine A. Christiansen, Gabriela G. Loots

**Affiliations:** 1 Biology and Biotechnology Division, Physical and Life Sciences Directorate, Lawrence Livermore National Laboratories, Livermore, California, United States of America; 2 School of Natural Sciences, University of California Merced, Merced, California, United States of America; 3 Department of Radiation Oncology, University of California Davis Medical Center, Sacramento, California, United States of America; 4 Department of Orthopaedic Surgery, University of California Davis Medical Center, Sacramento, California, United States of America; Thomas Jefferson University, UNITED STATES

## Abstract

Inhibitors of Wnt signaling have been shown to be involved in prostate cancer (PC) metastasis; however the role of Sclerostin (Sost) has not yet been explored. Here we show that elevated Wnt signaling derived from Sost deficient osteoblasts promotes PC invasion, while rhSOST has an inhibitory effect. In contrast, rhDKK1 promotes PC elongation and filopodia formation, morphological changes characteristic of an invasive phenotype. Furthermore, rhDKK1 was found to activate canonical Wnt signaling in PC3 cells, suggesting that SOST and DKK1 have opposing roles on Wnt signaling in this context. Gene expression analysis of PC3 cells co-cultured with OBs exhibiting varying amounts of Wnt signaling identified CRIM1 as one of the transcripts upregulated under highly invasive conditions. We found CRIM1 overexpression to also promote cell-invasion. These findings suggest that bone-derived Wnt signaling may enhance PC tropism by promoting CRIM1 expression and facilitating cancer cell invasion and adhesion to bone. We concluded that SOST and DKK1 have opposing effects on PC3 cell invasion and that bone-derived Wnt signaling positively contributes to the invasive phenotypes of PC3 cells by activating CRIM1 expression and facilitating PC-OB physical interaction. As such, we investigated the effects of high concentrations of SOST *in vivo*. We found that PC3-cells overexpressing SOST injected via the tail vein in NSG mice did not readily metastasize, and those injected intrafemorally had significantly reduced osteolysis, suggesting that targeting the molecular bone environment may influence bone metastatic prognosis in clinical settings.

## Introduction

Prostate cancer (PC) is the most frequently diagnosed cancer and the second leading cause of cancer-related deaths among men in the United States. If detected at early stages the prognosis is quite favorable; however, aggressive forms of metastatic PC spread primarily to the skeleton [1]. Bone tumors cause great pain, promote fractures, and ultimately represent the main cause of morbidity in patients suffering from advanced PC, with a 70% incidence documented by autopsies [2]. Most patients with advanced PC will experience major complications from bone metastases characterized by a mix of osteoblastic and osteolytic lesions, in which the osteoblastic component most often dominates. It has been hypothesized that the bone microenvironment is a major contributor to the PC metastatic process through the secretion of paracrine factors that attract, modulate, retain, and promote proliferation of PC cells in bone. Therefore knowledge of the local bone microenvironment is essential in understanding potential avenues for preventing the formation of secondary bone tumors in PC patients [3].

Wnt signaling has been shown to play many important roles during embryonic development, organ and tissue homeostasis, and bone biology. Activation of Wnt/β-catenin signaling occurs upon binding of Wnt ligands to the 7-transmembrane domain–spanning frizzled receptor coupled with low-density lipoprotein receptor–related protein 5 and 6 (LRP5/6) co-receptors. Intracellular signals are generated through a protein complex that includes Disheveled, Axin and Frat-1, which disrupt a GSK3-dependent protein complex that normally inhibits β-catenin function by targeting it for degradation. Stabilized β-catenin is then freed to translocate to the nucleus, where it interacts with other transcriptional activators such as T cell factor/lymphoid enhancer binding factor (TCF/LEF) to activate transcription. Binding of β-catenin subsequently displaces transcriptional co-repressors bound to TCF/LEF and recruits transcriptional co-activators like p300 and cAMP response element–binding protein [p300/CBP]) [4]. Wnt signaling is tightly regulated. One level of control is achieved through the interaction of secreted inhibitors with ligands or receptors to prevent ligand-receptor interaction. Ligand blocking proteins include Wnt inhibitory factor 1 (WIF1) and secreted frizzled-related proteins (Sfrp). Meanwhile, receptor inhibition is achieved by members of the sclerostin (SOST) and Dickkopf (DKK) families [4, 5], primarily through binding to LRP5/6 co-receptors.

Wnt malfunction has been implicated in various skeletal dysplasia and degenerative diseases. For example, loss- and gain-of function mutations in LRP5 cause either low or high bone mass [6, 7] and inactivation of SOST causes two hyperosteoses disorders: sclerosteosis [8, 9] and van Buchem's disease [10, 11]. A loss-of-function mutation in LRP6 is linked to an inherited disorder characterized by osteoporosis, coronary artery disease, and metabolic syndrome [12]. Furthermore, mutations in an intracellular regulator of β-catenin stability, WTX, cause osteopathia striata with cranial sclerosis [13, 14] and mutations in a Wnt co-receptor, FZD9, cause Williams–Beuren, a syndrome partially characterized by low bone density [15]. In addition, several genome wide association studies have identified polymorphisms in Wnt-signaling related genes linked to changes in bone mineral density [16–18]. Thus, even subtle alterations in the intensity, amplitude, and duration of Wnt signaling can interfere with skeletal development, bone remodeling, and bone regeneration.

Aberrant Wnt signaling is also commonly observed in cancer and the importance of this pathway stems from an initial observation that the tumor suppressor adenomatous polyposis coli (APC) downregulates β‐catenin. While loss of function mutations in Wnt signaling pathway genes are common to several cancers [19], the activation of Wnt signaling through the accumulation of nuclear β-catenin has also been documented for PC [5, 20]. Moreover, activators of Wnt signaling have been highly implicated in the invasive potential of metastatic cancer: Wnt5a [21], Wnt3a [22] and Wnt11 [23] have all been shown to alter cancer invasion and migration. Inhibitors of Wnt signaling also have been studied extensively. Frzb (PC [24], fibrosarcoma [25]) and WIF1 (PC [26] and urinary bladder cancer [27]), which modulate Wnt signaling by binding directly to Wnt ligands, are both known inhibitors of cancer invasion and migration. The most highly investigated (and contentious) effect of a Wnt signaling inhibitor on cancer invasion/metastatic potential is by the receptor-binding protein, DKK1. DKK1 has been shown to increase the invasive potential of esophageal cancer [28] and DKK1 antibodies have been used clinically as a target for passive immunotherapy [29]. However, DKK1 has also been shown to inhibit invasion and migration in colon cancer [30], breast cancer [31], and PC3 cells [32], indicating the complexity of studying this system *in vitro*. Interestingly, the effect of SOST on cancer invasion/metastasis has never been investigated. Therefore, in the current paper we focused on the role of SOST in PC invasion. In particular we examined the effects of osteoblast-derived Wnt signaling on PC to determine if the direct action of osteoblasts is required for modulating PC behavior.

## Materials and Methods

### Animals

All animal work was approved by the Lawrence Livermore National Laboratory (LLNL) IACUC committee. LLNL is an AAALAC accredited institution. *Sost* ^*KO*^ and *Lrp5* ^*KO*^ were previously described [33, 34]. KO and C57BL/6J (WT) mice were housed in standard conditions and all animal experiments were conducted according to NIH guidelines for animal use under approved IACUC protocols by the Lawrence Livermore National Laboratory.

### Cell lines and transfection conditions

PC3, DU145, C4-2Bm, LNCaP cells obtained from ATCC were cultured in DMEM; HPrEC were obtained from Lifeline Cell Technology and cultured in Lifeline's ProstaLife Medium. Expression plasmids (pCMV-DKK1, pCMV-SOST, pCMV-CRIM1) were generated by replacing the reporter gene of pmKate2-N (Evrogen, Moscow, Russia) with full-length cDNAs (Image Clones 3508222; 40009485; 8322423). Stable PC3 transfections of pCMV-DKK1, pCMV-SOST, pmKate2-vector were performed using Fugene 6 (Promega Corp., Madison, Wi.) as per the manufacturer’s instructions; positive clones were confirmed by qPCR or fluorescence. Mouse primary osteoblasts (OB) were collected enzymatically from calvaria of 4–5 day old pups similar to Bellows et al. 1986 [35]. Dissected calvaria free of periosteum was sequentially digested 5x at 37°C in 4 ml of collagenase solution (Collagenase 1, 0.625 mg/ml; Collagenase B, 1.875 mg/ml; CaCl_2_, 25 mM; in ddH_2_0 on ice) mixed with 1:2.5 media solution (DMEM/F-12, 0.1% BSA, 25 mM Hepes, 37°C), and fractions 2–5 were collected. Isolated OBs were cultured in DMEM/F-12 containing 10% FBS and 1% pen/strep.

### Canonical Wnt signaling assay

TOPFlash (0.9 μg) Wnt reporter plasmid (M50 Super 8x TOPFlash) and renilla luciferase control plasmid (0.1 μg) (RLTK) were transfected using 3 μl Fugene HD (Roche Applied Sciences, Indianapolis, IN) according to the manufacturer's protocol. After 24 hours, media in the cultures was changed to fresh media or media supplemented with recombinant protein. Co-cultures of isolated mouse osteoblasts cultured on 3 μm pore inserts (BD Falcon, cat# 3181) were also introduced to the PC3 cells at this time. Luciferase activities of both Super 8x TOPFlash and RLTK reporters were measured using a dual luciferase assay kit (Promega Corp.).

### Co-culture invasion assay

Invasion assays were performed using a modified Boyden chamber (BD bioscience). Matrigel (BD bioscience) was diluted 1:2.5 in ice-cold DMEM (serum-free) and 100 μl was transferred onto the upper chamber of an 8 μm pore insert (BD Falcon, cat# 3182) and allowed to solidify in an incubator for 2 h at 37°C. PC cells were plated on inserts at 2.5X10^4^, and OBs in 12-well plates at 5X10^4^; cells were allowed to seed ON. Following a 4h incubation in serum-free media, inserts were transferred into the 12-well plate containing OBs. Cells were counted at 20X on a Zeiss microscope after 48 h. DU145, C4-2Bm, LNCaP, and HPrEC cells were counted following staining in 1% Crystal Violet (Sigma) (30 min) followed by PBS washes. Invasion studies were accomplished using growth factors [rhSOST (R&D 1406-ST-025) 2.5-100ng/ml; rhDKK1 (R&D 5439-DK-010) 10-400ng/ml; TGFβ (R&D 240-B-002) 10ng/ml, WNT3A (R&D 5036-WN-010) 100ng/ml; PTH (R&D 7665-PT-050) 10nM and CRIM1 (R&D 1917-C-050)], in combination with PC3 cells, co-cultured for 48 h. PC3 cells were transiently transfected with pCMV-Crim1 plasmid using Fugene 6 during invasion and Crim1 expression was verified by qPCR [AGTTTCCAAGTCAGGATATGTGC (fwd); AGCATAACCCTCGATCAGAACA (rev) Crim 1 primers].

### Microarrays

Total RNA was extracted using an RNeasy Mini Kit, according to the manufacturer’s guidelines (QIAGEN). Samples were biotin labeled and hybridized on Human Genome U133 Plus 2.0 oligonucleotide arrays (PC3), according to the manufacturer’s recommendations (Affymetrix, Santa Clara, CA. USA). Data analysis was conducted as previously described [36].

### Immunostaining and actin staining

Immunofluorescent and phalloidin-TRITC staining were carried out on cells fixed in 4% paraformaldehyde for 15 min and permeabilized with 0.1% Triton X-100 for 5 min [anti-CRIM1 (HPA000556, Sigma) at 1:100; anti-Active-β-Catenin (05–665, Millipore) at 1:1000]. To stain for F-actin, cells were incubated with 50 μg/ml phalloidin-TRITC (Sigma) for 40 min. Imaging was done on Leica DM50000B and Zeiss LSM 510 Meta confocal microscopes. Maximum intensity projections were compiled using ImageJ (NCBI).

### Scanning electron-microscopy

PC3 cells (1X10^5^) were seeded onto 13 mm glass coverslips, then cultured for 48h in serum free media with or without recombinant protein. Cells were then fixed in 4% paraformaldehyde and processed as previously described [37].

### Xenograft

PC3, *PC3* ^*DKK1*^ and *PC3* ^*SOST*^ cells were injected into the tail vein (IV) or intrafemorally (IF) as previously described [38]. NSG (NOD.Cg-*Prkdc* ^*scid*^ *Il2rg* ^*tm1Wjl*^/SzJ) mice receiving PC cells IV (1X10^6^) were euthanized 10-weeks post-injection and tissues (lung, bone, heart, liver, kidney, spleen, and brain) were collected and examined for the presence of micro-tumors. Mice receiving PC cells IF (1x10^5^) were euthanized 4-weeks post-injection, femurs were dissected (N = 6), X-rayed, and bone volume of the proximal half of each femur was quantified by micro-computed tomography (μCT 35, SCANCO, Brüttisellen, Switzerland: energy 55 kVp, intensity 114 mA, integration time 900 ms, 10 μm nominal voxel size).

### Statistical Analysis

Significant differences were probed using ANOVA and paired t-test. *Post hoc* comparisons were made using Tukey’s [39]. Probability values <0.05 were taken as significant. Probability values shown in figures correspond to *p<0.05; **p<0.01; ***p<0.001.

## Results and Discussion

### SOST inhibits prostate cancer invasiveness

**Fig 1:**
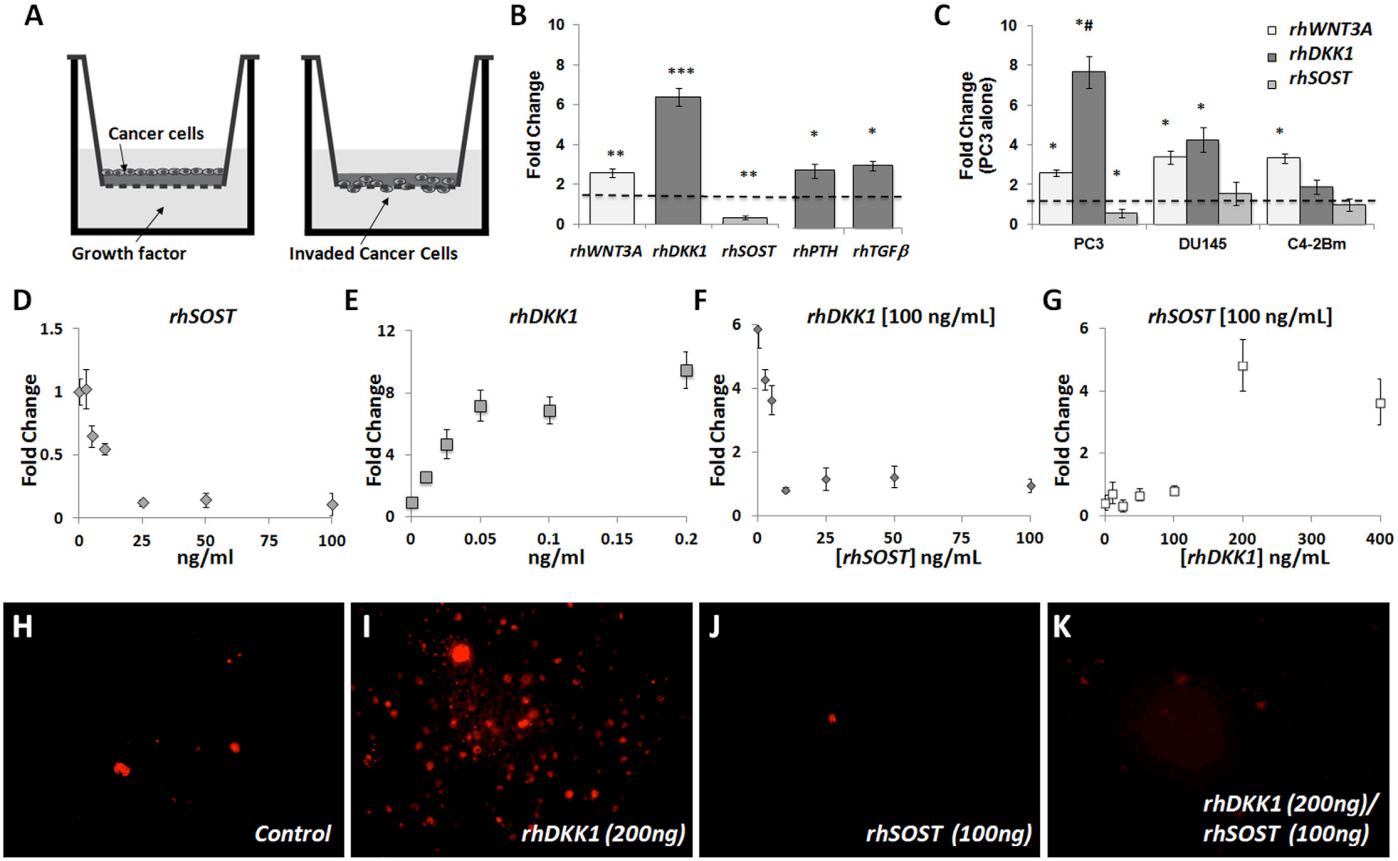
PC cell invasion. A, schematic representation of the invasion assay. B, invasive potential of PC3 cells following 48 hours of co-culture with recombinant proteins. C, the effect of rWNT3a, rDKK1, and rSOST on the invasion of PC3, DU145, and C4-2Bm. D-E, dose response curve of PC3 cells to rSOST or rDKK1. F-G, Dose response curves of PC3 cell invasion when either rDKK1 or rSOST was held constant and the other was incrementally increased. H-K, representative images of PC3-mKate cells following co-culture conditions. Results are expressed as fold change ± SEM. * P<0.5, ** P<0.1, *** P<0.01.

To investigate the effects of Wnt inhibitor Sclerostin (Sost) on the invasive potential of PC cells, we cultured PC3 cells with recombinant SOST (rhSOST) and measured their invasiveness into a matrigel (Fig 1A) relative to Wnt3a [32], parathyroid-related protein (PTHrP) [40], and transforming growth factor beta (TGFβ) [41, 42] treated PC3s. We also examined the Wnt antagonist DKK1, which is known to either promote or inhibit cancer invasion [28, 32, 43] in a context-dependent manner. Wnt3a, PTHrP and TGFβ increased PC3 invasive potential by ~3-fold (Fig 1B) and DKK1 increased invasion by 7-fold relative to PC3 alone and 3.5-fold relative to Wnt3a (Fig 1B and 1I). In contrast, rhSOST inhibited PC3 cell invasion by ~8-fold (Fig 1B and 1J). The increased invasiveness following WNT3a and DKK1 incubation was consistent in all three PC cell lines examined [(1) the highly invasive osteolytic lesion-inducing PC3; (2) the highly invasive osteoblastic lesion-inducing DU-145; (3) the invasive mixed phenotype C4-2Bm prostate cancer cell lines]. Moreover, rhSOST significantly inhibited the invasive potential of PC3 cells and blunted the Wnt3a-mediated invasiveness of DU145 and C4-2Bm cells (Fig 1C).

To determine rhSOST dose response and ranges of competitive inhibition we exposed PC3 cells to increasing concentrations of rhSOST and rhDKK1 and measured invasion. Increasing concentrations of rhSOST resulted in a dose-dependent decrease in invasion, with significant inhibition occurring as low as 2.5 ng/mL and saturation at 25 ng/mL (Fig 1D). Similarly, rhDKK1 also altered invasion in a dose-dependent manner; maximal invasiveness was observed at 50 ng/mL (Fig 1E). In order to investigate whether rhSOST could modulate rhDKK1-induced invasiveness we co-cultured PC3 cells with 100 ng/ml of rhDKK1 in combination with increasing amounts of rhSOST. This data indicated that rhSOST blunts rhDKK1-induced invasion even at the lowest doses (Fig 1F). The low-end rhSOST concentrations used were within the expected physiological range of serum SOST levels [44]. In contrast, increasing the rhDKK1 concentration was only able to overcome rhSOST-mediated inhibition at concentrations >200 ng/mL (Fig 1G), suggesting that SOST is a very potent inhibitor of PC3 invasion. These rhSOST inhibitory effects were also visualized in cultures of PC3 cells expressing the red fluorescent protein, pmKate2 (Fig 1J and 1K), where rhSOST dramatically inhibited DKK1-mediated PC3 invasion (Fig 1I and 1K).

### PC3 cells co-cultured with *Sost* ^*KO*^ osteoblasts show increased invasiveness

**Fig 2:**
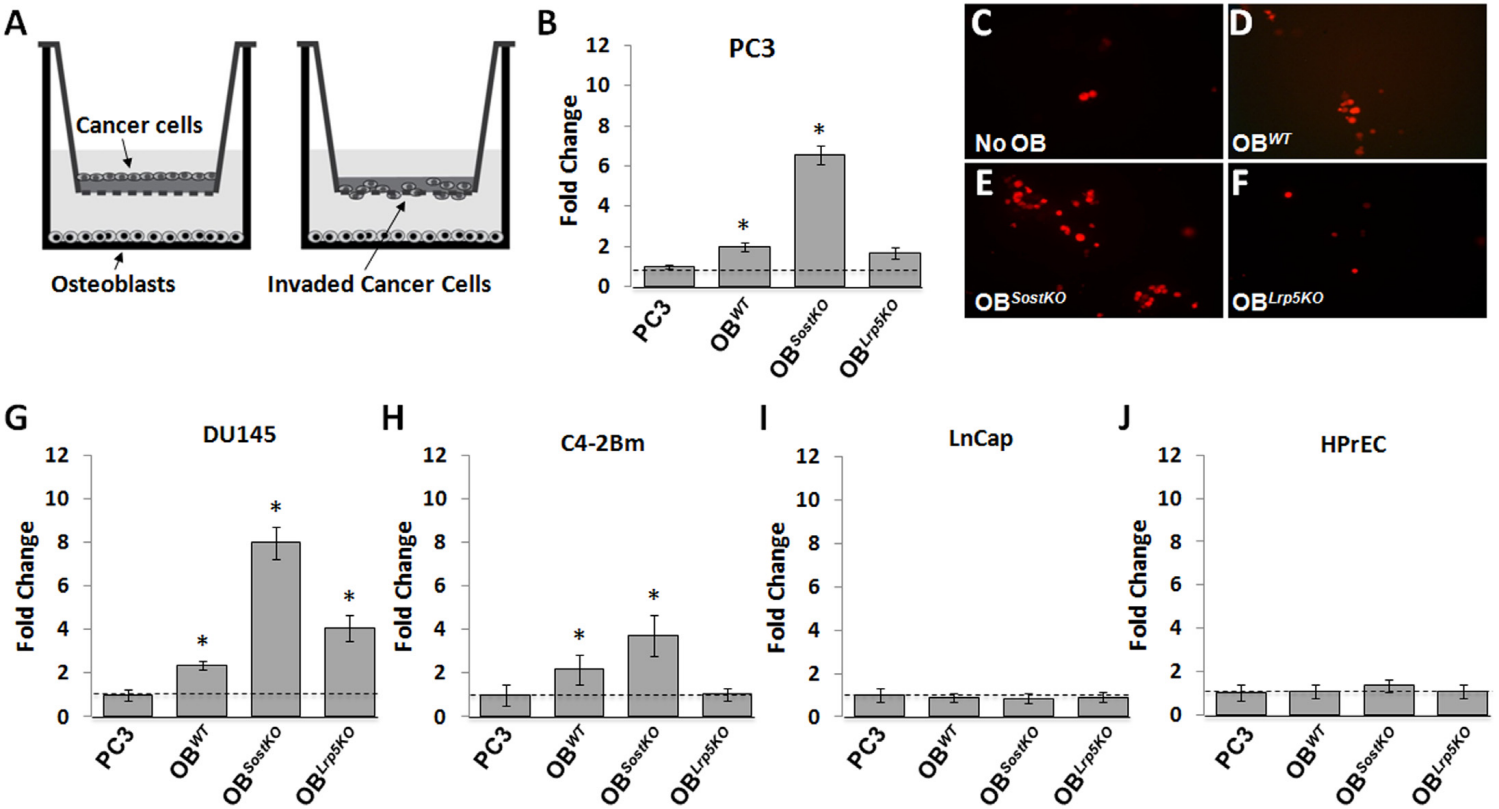
PC cell invasion towards osteoblasts isolated from neo-natal mice calvaria. A, schematic representation showing OBs grown on the bottom of the chamber. B, invasive potential of PC3 cells towards control OBs, OBs with elevated Wnt signaling, and OBs lacking Wnt signaling. C-F, representative images of PC3-mKate cells following co-culture conditions. G-J, invasive potential of PCa cells with different osteolytic potential towards each OB. Results are expressed as fold change ± SEM. * P<0.5, ** P<0.1, *** P<0.01.

Next we examined whether primary osteoblasts (OB) with varying levels of Wnt activity could modulate PC3 invasion. For these experiments, osteoblasts isolated from the calvaria of *Sost* knockout (KO) (OB^*SostKO*^), *Lrp5* KO (OB^*Lrp5KO*^) or C57BL/6N control mice (OB^*WT*^) were co-cultured without physical contact with PC3 cells (Fig 2A) and invasion was assessed both quantitatively and visually. Consistent with previous results, OB^*WT*^ co-culture induced a 2-fold increase in PC3 invasion. OBs lacking *Sost* increased PC3 invasion by >6-fold, while OBs lacking the *Lrp5* receptor abrogated this effect, decreasing invasion to the level of PC3 mono-cultures. These data indicate that low OB-derived Wnt signaling alone is sufficient to blunt the ability of OB^*WT*^ to promote PC3 invasion, *in vitro* (Fig 2B–2F). Similar to PC3 cells, DU145 and C4-2Bm cells also favored the OB^*WT*^ microenvironment and more readily invaded into the matrigel when co-cultured with OB^*SostKO*^ (Fig 2G and 2H). In addition, OB^*Lrp5KO*^ inhibited the invasiveness of C4-2Bm, but only slightly blunted the invasiveness of DU145 cells, suggesting that in the context of this co-culture the invasive potential of DU145 cells may not completely rely on Wnt signaling and may employ other molecular pathways (Fig 2G and 2H). Neither the relatively non-invasive LNCaP cell line of lymphatic metastasis origin nor the prostate epithelial cells (HPrEC) were affected by co-culture with OBs (Fig 2I and 2J). These data indicate that osteophilic PC cell lines are readily modulated by the bone microenvironment and that small changes in the amount of OB-derived Wnt signaling can alter their invasive potential, *in vitro*. In contrast, LNCaP cells which do not readily metastasize to the bone are not affected by modulated levels of Wnt signaling in neighboring osteoblasts, suggesting that prostate cancer metastasis to bone requires a mutual response promoted by bone-cancer interaction.

### rhDKK1 activates Wnt signaling in PC3 cells

**Fig 3:**
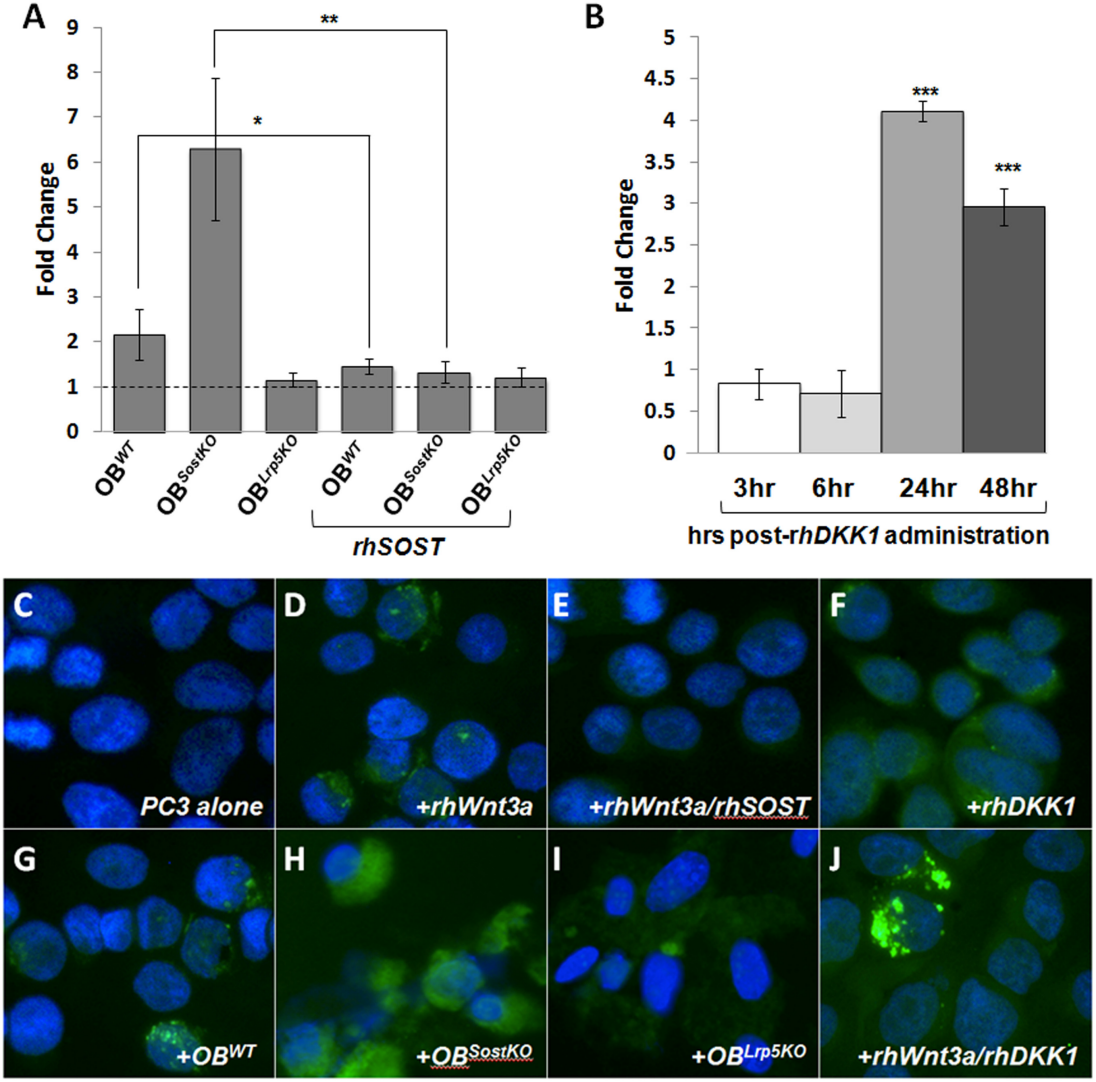
Downstream Wnt activity following 48hrs of co-culture. A, TopFlash reporter Luciferase assay in PC3 cells co-cultured with OBs. B, TopFlash reporter assay in PC3 cells co-cultured with DKK1. C-J, activated β-catenin (ABC) immunohistochemistry in PC3 cells under multiple conditions; green (ABC), blue (DAPI). Results are expressed as fold change ± SEM. * P<0.5, ** P<0.1, *** P<0.01.

DKK1 and SOST are known inhibitors of Wnt signaling in bone [45], yet they have opposing effects on PC invasion (Fig 1B). We next investigated the state of Wnt signaling in this co-culture invasion model. TopFlash reporter expressing secreted Luciferase was transfected into PC3 cells (PC3^*CLucTF*^). Wnt3a enhanced relative luminescence in a dose and time dependent manner (S1 Fig). Co-culture of PC3^*CLucTF*^ with OB^*WT*^ or OB^*SostKO*^ significantly increased reporter activity, an effect blocked by rhSOST (Fig 3A). Co-culture with OB^*LRP5KO*^ had no significant effect on Wnt signaling. This data suggests that PC cells may rely on the Wnt signaling activity of the surrounding bone milieu and loss or gain of Wnt signaling in the bone could have profound effects on the Wnt signaling activity within the neighboring/invading cancer cells. Although initial rhDKK1 treatment repressed Wnt reporter activity, it significantly increased PC3^*CLucTF*^ luciferase activity at 24 hours post rhDKK1 administration and later time points (Fig 3B).

To corroborate the mechanisms involved in rhDKK1 Wnt agonist activity on the TopFlash reporter transgene, we next examined levels of activated β-catenin (ABC) in PC3 cells following a 48-hour co-culture, via immunocytochemistry. PC3 cells alone expressed insignificant amounts of ABC (Fig 3C). Administration of rhWnt3a enhanced ABC signal within both the cytoplasm and in the nucleus of the PC3 cells (Fig 3D) and this effect was perceptibly inhibited by rhSOST co-administration (Fig 3E). Consistent with the PC3^*CLucTF*^ data, rhDKK1 increased ABC in PC3 cells (Fig 3F). PC3 cells co-cultured with OB^*WT*^ also increased ABC levels (Fig 3G), an effect vigorously enhanced in OB^*SostKO*^ co-cultures (Fig 3H) and diminished in OB^*LrpKO*^ co-cultures (Fig 3I). Furthermore co-administration of both Wnt3A and DKK1 to PC3 cells resulted in hyper activation of ABC in a subset of PC3 cells, suggesting a synergistic and additive effect on the activation of canonical Wnt signaling in these cells. These results highlight the importance of the bone microenvironment on the invading prostate cancer cells, and the ability of bone-derived Wnt signaling to activate Wnt signaling in these cells. The correlation between high DKK1 levels and increased β-catenin levels is consistent with previously reported data where patient-derived tumors with elevated DKK1, β-catenin, or both had were more likely to have a poor hepatocellular carcinomas prognosis [46].

### Filopodia formation is increased in DKK1 and CRIM1 treated cells

**Fig 4:**
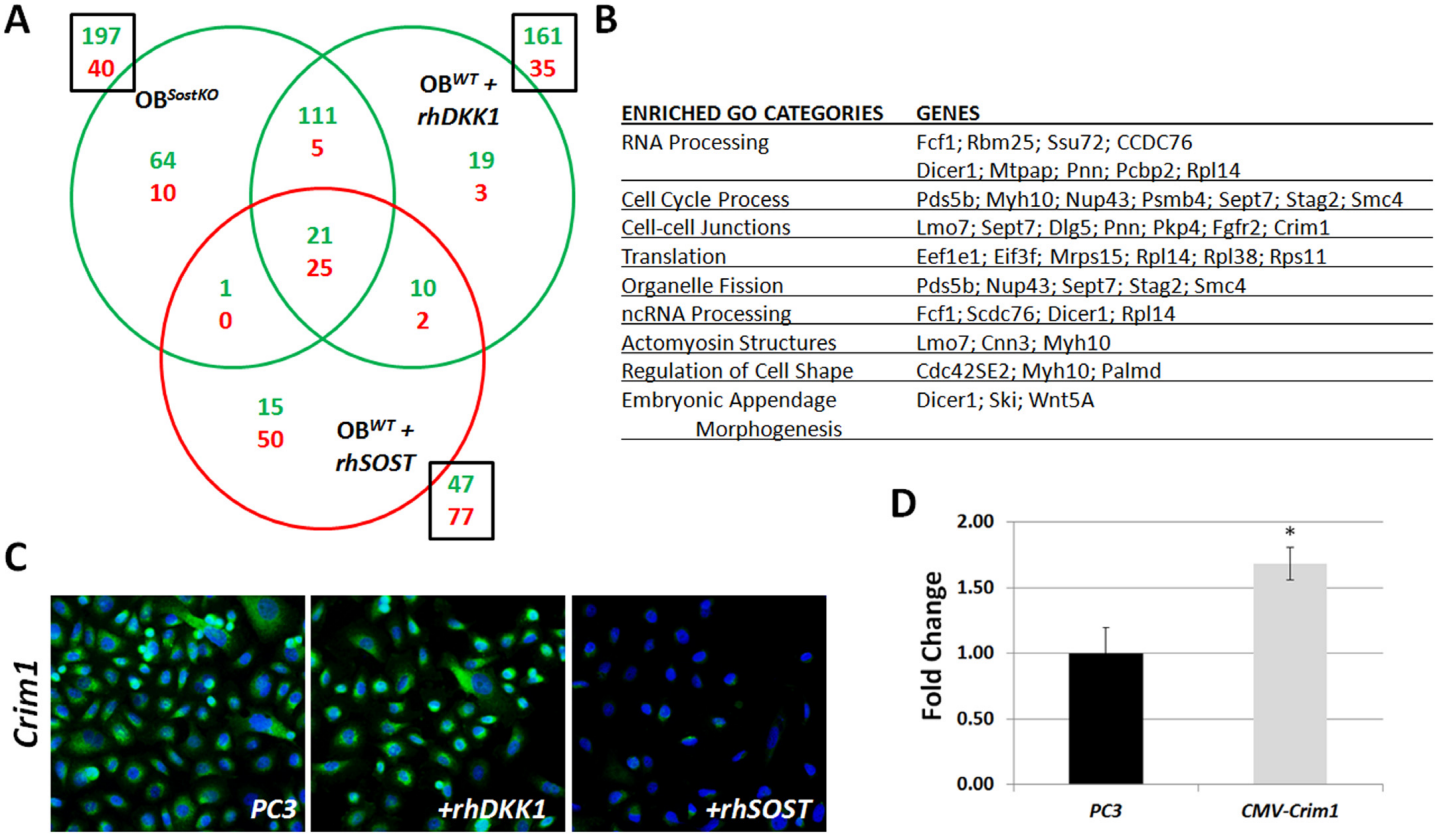
Molecular changes in invasive PC3 cells. A, microarray analyses of PC3 cells co-cultured with OB^*SostKO*^, OB^*WT*^+ rhDKK1, and OB^*WT*^ + rhSOST as compared to monocultures; overlay representation of upregulated (green) and downregulated (red) genes. B, representative list of upregulated transcripts. C, representative images of CRIM1 protein expression modulation by rhDKK1 and rhSOST; green (CRIM1), blue (DAPI). Results are expressed as fold change ± SEM. * P<0.5, ** P<0.1, *** P<0.01. D, PC3 transfected with a CMV-Crim1 expression vector were significantly more invasive then PC3 cells (*p*-value 0.006).

Having established that Sost-deficient OBs or OBs treated with rhDKK1 increase PC3 invasiveness we further explored the shared molecular changes in these co-cultured PC3 cells through transcription analysis. We compared gene expression changes in PC3 cells co-cultured with (1) OB^*SostKO*^, (2) OB^*WT*^+ rhDKK1, and (3) OB^*WT*^ + rhSOST (Fig 4A). We found 132 upregulated genes and 30 downregulated genes in both OB^*SostKO*^ and OB^*WT*^+ rhDKK1 co-cultured with PC3 cells (S1 Table). Among these transcripts 21 were upregulated and 25 were downregulated in OB^*WT*^ + rhSOST PC3 co-cultures (S2 Table). The upregulated transcripts in the highly invasive PC3 cells were enriched for molecules that have been shown to be involved in regulating cell shape, cell migration/motility, and cell adhesion. Upregulated genes included: *sept7* [47], *myo10* [48], *PKP4* [49], *cnn3* [50], *Fgfr2* [51], and *crim1* [52] (Fig 4B). Of particular interest, the cysteine-rich motor neuron protein 1 or Crim1 is a single-pass (type 1) transmembrane protein that has recently been shown to complex with β-catenin and cadherins. Crim1 loss of function experiments in *Xenopus* revealed Crim1 to be critical for cell-cell adhesion during neural development [52]. Elevated levels of β-catenin (Fig 3H) accompanied by elevated levels of Crim1 (S1 Table) in co-cultured PC3 cells led us to hypothesize that Crim1 may be upregulated by bone-derived Wnt signaling and may be involved in promoting cell invasion and or cell adhesion.

To determine whether CRIM1 protein expression is modulated by rhDKK1 and rhSOST, consistent with the gene expression data, we quantified the relative fluorescence intensity of CRIM1 in PC3 cell treated with rhDKK1 and rhSOST. We found the rhDKK1 treated cells to be 20% brighter [0.345 (PC3+hrDKK1) vs 0.285 (PC3) mean fluorescence/cell] while the rhSOST treated PC3 cells were 85% dimmer [0.038 (PC3+hrSOST) vs 0.285 (PC3) mean fluorescence/cell] than untreated PC3 cells (Fig 4C). Furthermore overexpression of CRIM1 protein [>12 fold overexpression as determined by qPCR], in PC3 transfected with a CMV-Crim1 constructed showed a significant increase in invasion (*p*-value = 0.006), in the transwell assay (Fig 4D).

**Fig 5:**
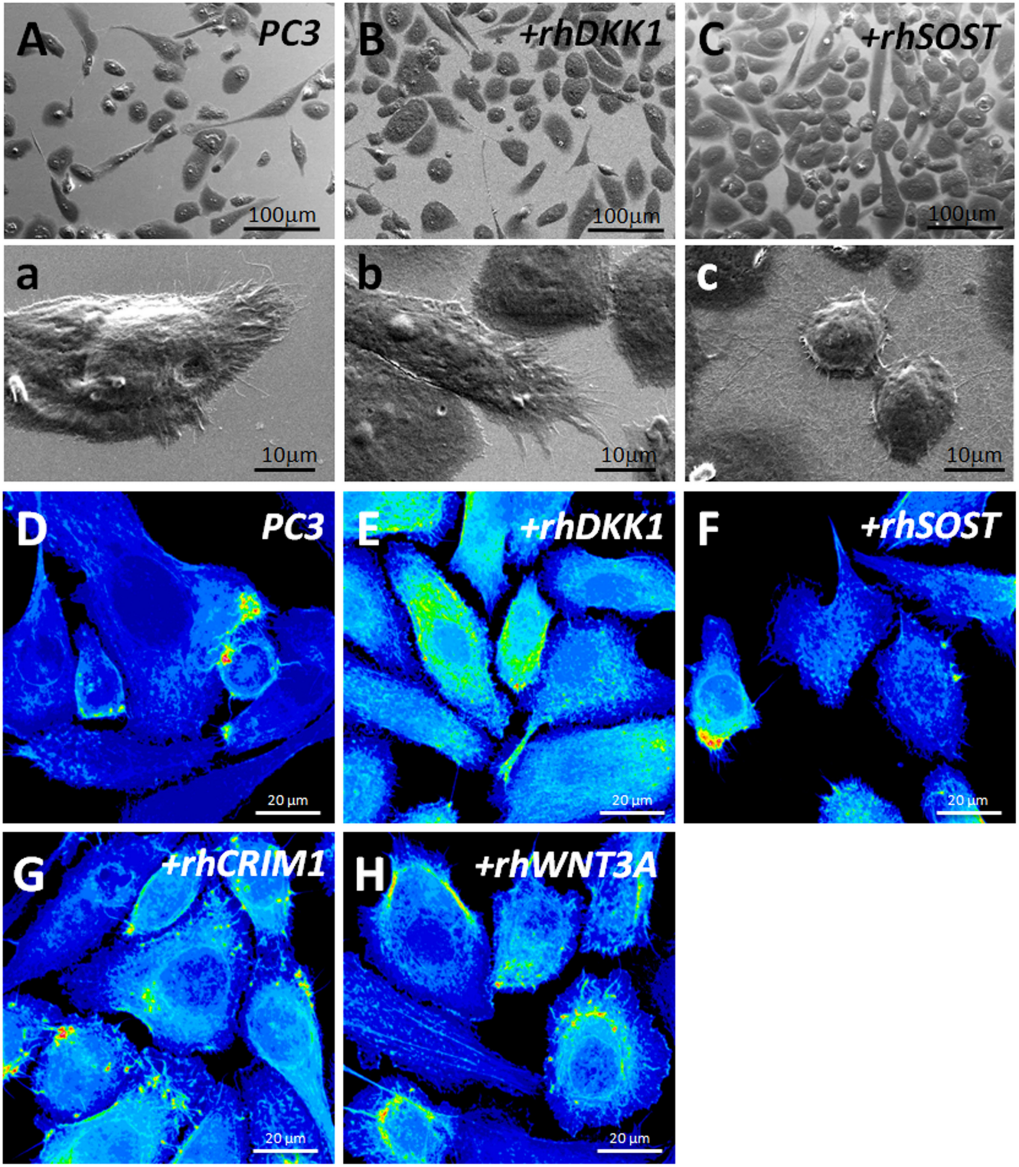
Morphological changes in PC3 cells. A-C, Representative SEM images (1500x) of PC3 cells treated with rhDKK1 (B, b), or rhSOST (C, c). D-H, Immunofluorescence staining of PC3 cells treated with rhDKK1 (E), rhSOST (F), rhCRIM1 (G) and rhWNT3A (H); anti–CRIM1 (green) and rhodamine-conjugated phalloidin (blue).

To investigate whether the effects of DKK1 and CRIM1 on cancer cell invasion and metastasis are associated with actin filaments, we examined morphological changes in PC3 cells treated with rhDKK1 and rhSOST. *PC3* cells treated with DKK1 were elongated, displaying fibroblast-like morphology with many more filopodia projections as compared to either untreated or rhSOST treated PC3 cells (Fig 5A–5C), which were more rounded and had fewer filopodia. Moreover, both untreated and rhSOST treated PC3 cells adhered less strongly to the plastic surface and tended to be more clumped together forming aggregates. Morphological appearance was consistent with the underlying cytoskeletal organization; phalloidin staining showed enhanced actin organization in rhDKK1 and rhWNT3A treated PC3 cells, relative to PC3 and rhSOST treated cells (Fig 5D–5H). Similarly, the exogenous addition of a secreted form of CRIM1 also promoted the formation of lamellipodia and filopodia, as visualized by phalloidin–stained actin in PC3 cells treated with CRIM1. Collectively, these findings indicate that both DKK1 and CRIM1 promote filopodia formation in highly invasive PC3 cells and that this process is associated with the reorganization of actin filaments.

### SOST overexpression inhibits metastasis and blunts osteolysis

**Fig 6:**
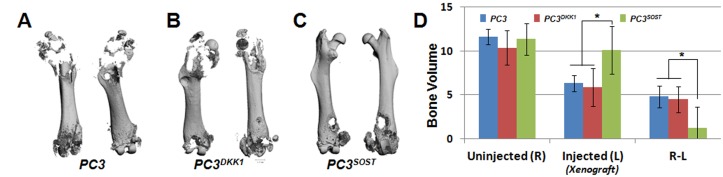
SOST reduces *PC3*-mediated osteolysis in xenograft derived tumor lesions. Representative femur micro-CT scans from *PC3* (A), *PC3* ^*DKK1*^ (B) and *PC3* ^*SOST*^ (C) injected NSG mice (N = 6/group). Bone volume was quantified for both PC injected and uninjected contralateral femurs, and relative bone loss due to osteolysis was calculated for each group by subtracting the injected (L) from the uninjected (R) values (D). *PC* ^*SOST*^ injected femurs experienced significantly less bone loss due to advanced osteolytic lesions (*p<0.05).

To determine whether SOST impacts metastasis, NSG mice we injected intravenously with PC3, PC3^*DKK1*^ or PC3^*SOST*^ and tissues were examined for the presence of tumors 10 weeks post injection. Intravenous delivery of PC3 cells resulted in an 80% tumor rate (8/10 mice) with lung and kidney being the most frequent sites of metastasis. PC3^*DKK1*^ cells behaved highly similar to PC3 cells, while PC3^*SOST*^-injected mice had significantly lower rates of metastasis, with only 1/9 mice (spleen) showing visible macroscopic metastasis at 10-weeks post-injection (Table 1). These data suggested that SOST-induced inhibition of Wnt signaling significantly reduces PC3 invasion and metastasis, *in vivo*. Next we examined whether overexpression of *SOST* in PC3 cells reduces osteolytic tumor formation. Ten NSG mice per group received 5x10^5^ PC3, PC3^*DKK1*^ or PC3^*SOST*^ cells intrafemorally. Four weeks post injections bone volume was quantified using micro-CT. While all three groups showed bone loss and damage at the distal site where the needle was inserted to deliver the cancer cells, the scans showed dramatic loss of bone in the proximal femur of PC3 and PC3^*DKK1*^, but significantly less bone loss in PC3^*SOST*^ injected femurs (Fig 6A–6C). To assess bone loss differences due to osteolysis we subtracted the bone volume of the PC injected femur from the contralateral uninjected femur and found PC3^SOST^ cells to induce significantly less bone loss than PC3 or PC3^*DKK1*^. These findings suggest that the overexpression of SOST in PC3 cells blunts osteolysis and reduces the tumor formation rate.

**Table 1:** Micro-metastatic distribution 10-weeks post intravenous injection of PC3 cells.

		% Animals with Tumors	Liver	Lung	Spleen	Kidney	Heart
Sham	(N = 5)	0	0	0	0	0	0
PC3	(N = 10)	80%	0	5	1	5	0
PC3^*DKK1*^	(N = 9)	67%	0	4	0	1	0
PC3^*SOST*^	(N = 9)	18%	0	0	1	0	0

## Discussion

In this report we examined the molecular mechanisms modulated by Wnt signaling that may contribute to the well-documented high tropism of prostate cancer to bone, during metastasis. We hypothesized that the bone derived Wnt signaling triggers gene expression changes in the cancer cells that enhances their attachment and homing to bone as well as promotes bone malignancies. To test this hypothesis, we exposed PC cells to Wnt antagonists DKK1 and SOST, and found DKK1 to promote, and SOST to inhibit PC invasion. Furthermore, osteoblasts isolated from *Sost* knockout mice had a potent positive effect on PC invasion; an effect that was dramatically blunted by the addition of recombinant SOST protein. In the last decade, DKK1’s role in cancer has been controversial. In some instances, DKK1 was found to be highly expressed and to correlate with poor overall and disease-free survival. For example, it has been suggested that DKK1 could be used as a prognostic marker and therapeutic target for hepatocellular carcinoma [43]. However, in other instances, DKK1 was found to be repressed in certain colon cancers and it was suggested that DKK1 might act as a tumor suppressor gene [53]. Here we find DKK1 to (1) promote PC3 invasion, *in vitro*, (2) activate TOPFLASH, a canonical Wnt signaling reporter, and (3) upregulate β-catenin, suggesting that in this context, DKK1 acts as a Wnt agonist. In sharp contrast we found SOST to (1) inhibit PC3 invasion, *in vitro*, (2) repress TOPFLASH expression, and (3) inhibit β-catenin protein expression, consistent with its described role in bone as a Wnt antagonist. Treatment of PC3 cells with DKK1 increased their invasion into the matrigel, correlating high levels of DKK1 expression with a poor cancer prognosis.

Future studies that would potentially elucidate the context-dependent role of DKK1 in cancer progression and metastasis would be: dissecting out the cell- and non-cell autonomous effects of DKK1, and using genetics to characterize the correlative vs. the causative effects of DKK1. Furthermore, we have yet to determine if DKK1 and SOST are similarly processed by cancer cells. It may be that SOST only blocks the receptor as the cell surface, while DKK1 is internalized and has additional signal transaction effects downstream of the WNT-receptors. Since the bone microenvironment is uniquely favorable to PC metastasize, and regulators of bone formation such as DKK1 modulate this process, a better understanding of the molecular interactions that promote tumor cells to home to the bone is key to understanding bone metastasis and exploring preventive treatment. Since SOST’s role in cancer has been minimally explored, it also remains to be elucidated whether this molecule, similar to DKK1 has context-dependent effects on cancer.

Cell morphogenic changes and cell motility are essential for the epithelial to mesenchymal (EMT) as well as mesenchymal to epithelial transitions (MET), processes that are required for intravasation [the exit from the primary tumor site and entry into circulation] and extravasation [the exit from the blood stream into secondary site]. These steps are one of the first to occur during the process of metastasis; tumor cells that are shed into the circulation are the root of distant macrometastases. We find that DKK1-treated PC3 cells dramatically alter their morphology, undergoing cytoplasmic and membranous changes associated with formation of filopodia structures know to aid cell movements. Furthermore, we found *crim1* to be upregulated in highly invasive cells, but not in non-invasive cells. This protein has been shown to be critical for cell movement and cell adhesion during neural plate development. In a very elegant study, Ponferrada et al. knocked down CRIM1 in Xenopus embryos using morpholinos and showed that CRIM1 is essential for the formation of the nervous system. By forming a complex with β-catenin and cadherins, CRIM1 participates in the formation of adhesion junctions, and in the absence of CRIM1 these proteins fail to localize to these junctional complexes. Additionally, they showed that CRIM1 alone did not mediate cell-cell adhesion, however it was essential for the formation and stabilization of cadherin-dependent adhesion complexes. This led us to speculate that once a PC cell arrives to the bone, the microenvironment rich in Wnt signaling facilitates molecular changes in cancer cells, including the upregulation of CRIM1 expression. Elevated levels of CRIM1 subsequently may promote the formation and stabilization of cadherin-dependent adhesion complexes, which may mediate cell-cell physical contact between bone and cancer cells. Because CRIM1 is significantly upregulated in both PC3 cells co-cultured with OB derived from *Sost* knockout mice and PC3 cells co-cultured with *WT* OBs treated with DKK1, we conclude that *crim1* upregulation is in part responsible for the increased invasiveness of PC3 cells. Moreover, since *crim1* overexpression increases cell-cell adhesion, our findings also suggest that CRIM1 may be involved in promoting bone metastasis and may in part be responsible for the inherent affinity of certain strains of cancer to form bone tumors.

Cell-cell adhesion among animal cells is a dynamic process, where cells may constantly be involved in movement without losing cell-cell contact. Molecules involved in cell-cell contact or motility translocate along the cell membrane and contribute to cellular polarity. In addition, cells also interact with extracellular matrix (ECM), and in certain instances, the interaction with the ECM may contribute to junctional instability, morphogenesis and cell-cell contact. On one hand, CRIM1 may play a role in regulating cadherin-catenin junctional stability; however we have yet to show how CRIM1 affects attachment to a mineral substrate and ECM characteristics of bone. This studies uses purified as surrogates for the bone microenvironment, future studies would help determine how PC3-OB cell-cell interactions are influenced by mineralization and specific collagenous substrates found in bone. Furthermore, most bone metastatic lesions are promoted by enhanced osteoclast activity [54–56], and Wnt signaling in osteoblasts is primarily anabolic, therefore we have to reconcile how an anabolic bone program in *Sost* ^*KO*^ osteoblasts enhances invasion and osteolysis. In conclusion, consistent with previously published preclinical experiments that support the inhibition of Wnt signaling as a potential mechanism for hindering tumor cell growth, tumor survival, and metastasis, in the current study we show that sclerostin, a Wnt antagonist, has the potential to inhibit prostate cancer invasion, *in vitro*, and to reduce the incidence of macroscopic metastases and osteolysis in NSG mice, *in vivo*.

## Supporting Information

S1 Fig Wnt3a enhanced relative luminescence in a dose and time dependent manner. PC3 cells were transfected with TOPFLASH and 3 concentration of rhWnt were added immediately after transfection [100, 50, 25 ng/ml]. Luminescence was quantified at 3, 6, 24 and 48 hours post transfection.
(TIF)

S1 Table Highly Invasive Genes. Transcripts that are significantly changed in both in *Sost* ^*KO*^ and DKK1 co-cultured PC3 cells, but are not significantly changed (they have a *p*-value greater than 0.05 or Fold change less than 2) in PC3 cells treated with rhSOST.
(DOCX)

S2 Table Low invasive Genes. Transcripts that are significantly changed in PC3 cells treated with rhSOST, but not significantly changed in PC3 cells co-cultured with *Sost* ^*KO*^ osteoblasts and DKK1.
(DOCX)
