# Supplementary figures and images for: SOST Inhibits Prostate Cancer Invasion
Source: PLoS One. 2015 Nov 6;10(11):e0142058. doi: 10.1371/journal.pone.0142058 (PMC4636315; doi:10.1371/journal.pone.0142058)

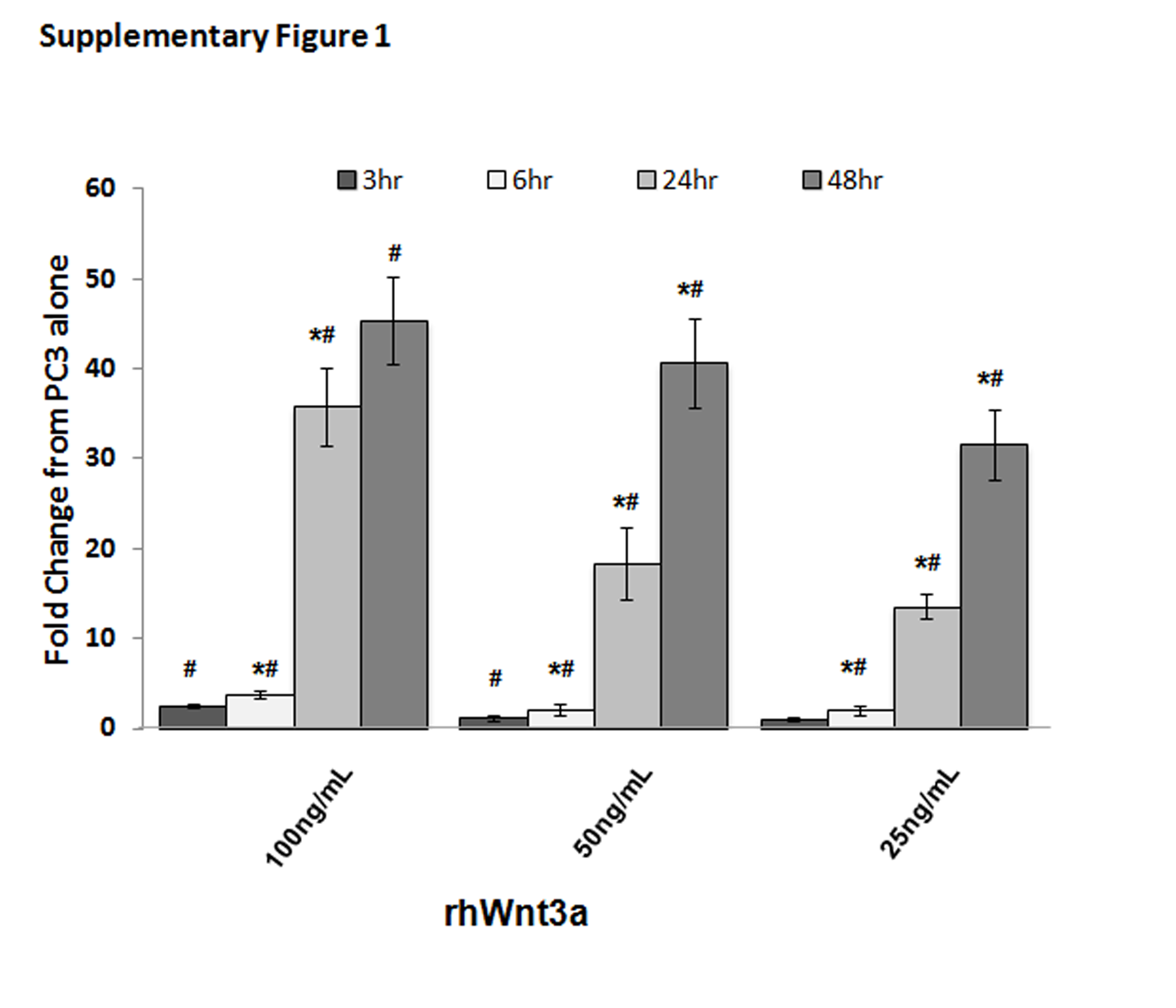

Supplement: S1 Fig — PC3 cells were transfected with TOPFLASH and 3 concentration of rhWnt were added immediately after transfection [100, 50, 25 ng/ml]. Luminescence was quantified at 3, 6, 24 and 48 hours post transfection. (TIF) [file pone.0142058.s001.tif]
