# Supplementary material for: SOST Inhibits Prostate Cancer Invasion
Source: PLoS One. 2015 Nov 6;10(11):e0142058. doi: 10.1371/journal.pone.0142058 (PMC4636315; doi:10.1371/journal.pone.0142058)
Supplement: S1 Table — Transcripts that are significantly changed in both in Sost KO and DKK1 co-cultured PC3 cells, but are not significantly changed (they have a p-value greater than 0.05 or Fold change less than 2) in PC3 cells treated with rhSOST. (DOCX) [file pone.0142058.s002.docx]

**S1 Table.** **Highly Invasive Genes**. Transcripts that are significantly changed in both in *Sost^KO^* and DKK1 co-cultured PC3 cells, but are not significantly changed (they have a *p*-value greater than 0.05 or Fold change less than 2) in PC3 cells treated with rhSOST

| **UP-REGULATED TRANSCRIPTS** | | | **Fold Change** | |
| --- | --- | --- | --- | --- |
| **No** | **Symbol** | **Gene name** | ***Sost^KO^*** | **DKK1** |
| 1 | SEL1L | sel-1 suppressor of lin-12-like (C. elegans) | 5.21 | 4.39 |
| 2 | Sept7 | septin 7 | 4.96 | 3.86 |
| 3 | PRRC2C | proline-rich coiled-coil 2C | 4.77 | 4.65 |
| 4 | C18orf54 | chromosome 18 open reading frame 54 | 4.56 | 3.67 |
| 5 | SKI | v-ski sarcoma viral oncogene homolog (avian) | 4.30 | 3.37 |
| 6 | MZT1 | mitotic spindle organizing protein 1 | 3.93 | 2.84 |
| 7 | PRPS2 | phosphoribosyl pyrophosphate synthetase 2 | 3.87 | 3.44 |
| 8 | SCD | stearoyl-CoA desaturase (delta-9-desaturase) | 3.74 | 3.27 |
| 9 | ETV5 | ets variant 5 | 3.72 | 3.01 |
| 10 | UBE2H | ubiquitin-conjugating enzyme E2H | 3.68 | 2.76 |
| 11 | ARGLU1 | arginine and glutamate rich 1 | 3.48 | 3.45 |
| 12 | ZNF652 | zinc finger protein 652 | 3.46 | 3.26 |
| 13 | PDP2 | pyruvate dehyrogenase phosphatase catalytic subunit 2 | 3.39 | 3.33 |
| 14 | DLG5 | discs, large homolog 5 (Drosophila) |  | 3.25 |
| 15 | LRRC16A | leucine rich repeat containing 16A | 3.24 | 2.60 |
| 16 | MARCKS | myristoylated alanine-rich protein kinase C substrate | 3.22 | 2.80 |
| 17 | AP1S2 | adaptor-related protein complex 1, sigma 2 subunit | 3.21 | 3.22 |
| 18 | MAGT1 | magnesium transporter 1 | 3.19 | 3.11 |
| 19 | DCBLD2 | discoidin, CUB and LCCL domain containing 2 | 3.13 | 2.64 |
| 20 | FEM1A | fem-1 homolog a (C. elegans) | 3.11 | 2.22 |
| 21 | GPR126 | G protein-coupled receptor 126 | 3.07 | 2.75 |
| 22 | CSNK2A1 | casein kinase 2, alpha 1 polypeptide | 3.07 | 2.79 |
| 23 | BOLA2 | bolA homolog 2 (E. coli) | 2.97 | 3.00 |
| 24 | GNL3L | guanine nucleotide binding protein-like 3 (nucleolar)-like | 2.96 | 2.88 |
| 25 | MYO10 | myosin X | 2.95 | 2.76 |
| 26 | PDS5B | PDS5, regulator of cohesion maintenance, homolog B (S. cerevisiae) | 2.94 | 2.35 |
| 27 | PPM1A | protein phosphatase, Mg2+/Mn2+ dependent, 1A | 2.94 | 2.46 |
| 28 | RPS11 | ribosomal protein S11 | 2.91 | 2.74 |
| 29 | CBX5 | chromobox homolog 5 | 2.90 | 3.17 |
| 30 | SLMO2 | slowmo homolog 2 (Drosophila) | 2.90 | 2.70 |
| 31 | PCBP2 | poly(rC) binding protein 2 | 2.89 | 2.57 |
| 32 | TMEM106B | transmembrane protein 106B | 2.87 | 2.71 |
| 33 | WNT5A | wingless-type MMTV integration site family, member 5A | 2.86 | 2.63 |
| 34 | WSB2 | WD repeat and SOCS box containing 2 | 2.80 | 2.63 |
| 35 | RASL11A | RAS-like, family 11, member A | 2.78 | 2.61 |
| 36 | CPNE8 | copine VIII | 2.76 | 2.60 |
| 37 | TGIF2 | TGFB-induced factor homeobox 2 | 2.75 | 2.29 |
| 38 | PSMB4 | proteasome (prosome, macropain) subunit, beta type, 4 | 2.75 | 2.26 |
| 39 | CNN3 | calponin 3, acidic | 2.70 | 2.57 |
| 40 | ARHGAP21 | Rho GTPase activating protein 21 | 2.69 | 2.06 |
| 41 | NUCKS1 | nuclear casein kinase and cyclin-dependent kinase substrate 1 | 2.62 | 2.71 |
| 42 | TMEM106C | transmembrane protein 106C | 2.61 | 2.36 |
| 43 | DICER1 | dicer 1, ribonuclease type III | 2.61 | 2.28 |
| 44 | CDC42SE2 | CDC42 small effector 2 | 2.58 | 2.26 |
| 45 | LMO7 | LIM domain 7 | 2.58 | 2.42 |
| 46 | CXorf56 | chromosome X open reading frame 56 | 2.56 | 2.17 |
| 47 | PGGT1B | protein geranylgeranyltransferase type I, beta subunit | 2.56 | 2.18 |
| 48 | HEATR3 | HEAT repeat containing 3 | 2.55 | 2.36 |
| 49 | MRPS15 | mitochondrial ribosomal protein S15 | 2.54 | 2.63 |
| 50 | BBIP1 | BBSome interacting protein 1 | 2.54 | 2.29 |
| 51 | ZNF420 | zinc finger protein 420 | 2.54 | 2.41 |
| 52 | HSP90B1 | heat shock protein 90kDa beta (Grp94), member 1 | 2.54 | 2.72 |
| 53 | MYH10 | myosin, heavy chain 10, non-muscle | 2.53 | 2.51 |
| 54 | JAG1 | jagged 1 | 2.53 | 2.37 |
| 55 | GOLIM4 | golgi integral membrane protein 4 | 2.52 | 2.25 |
| 56 | PKP4 | plakophilin 4 | 2.48 | 2.04 |
| 57 | RASSF3 | Ras association (RalGDS/AF-6) domain family member 3 | 2.45 | 2.13 |
| 58 | NIPAL3 | NIPA-like domain containing 3 | 2.43 | 2.16 |
| 59 | PDK3 | pyruvate dehydrogenase kinase, isozyme 3 | 2.43 | 2.46 |
| 60 | MGA | MAX gene associated | 2.42 | 2.06 |
| 61 | SUN1 | Sad1 and UNC84 domain containing 1 | 2.41 | 2.51 |
| 62 | RBM25 | RNA binding motif protein 25 | 2.41 | 2.35 |
| 63 | PNN | pinin, desmosome associated protein | 2.40 | 2.31 |
| 64 | PGK1 | phosphoglycerate kinase 1 | 2.39 | 2.56 |
| 65 | RPL14 | ribosomal protein L14 | 2.39 | 2.43 |
| 66 | EP400 | E1A binding protein p400 | 2.39 | 2.16 |
| 67 | UQCRC2 | ubiquinol-cytochrome c reductase core protein II | 2.37 | 2.14 |
| 68 | SLFN13 | schlafen family member 13 | 2.34 | 2.20 |
| 69 | RWDD2B | RWD domain containing 2B | 2.33 | 2.08 |
| 70 | PPAP2B | phosphatidic acid phosphatase type 2B | 2.33 | 2.43 |
| 71 | OTUD1 | OTU domain containing 1 | 2.33 | 2.28 |
| 72 | PKN2 | protein kinase N2 | 2.32 | 2.67 |
| 73 | FOXA1 | forkhead box A1 | 2.31 | 2.11 |
| 74 | C6orf48 | chromosome 6 open reading frame 48 | 2.29 | 2.14 |
| 75 | RAP2A | RAP2A, member of RAS oncogene family | 2.29 | 2.16 |
| 76 | C15orf29 | chromosome 15 open reading frame 29 | 2.28 | 2.15 |
| 77 | STAG2 | stromal antigen 2 | 2.28 | 2.23 |
| 78 | EEF1E1 | eukaryotic translation elongation factor 1 epsilon 1 | 2.27 | 2.21 |
| 79 | MRPS5 | mitochondrial ribosomal protein S5 | 2.27 | 2.11 |
| 80 | LTBP1 | latent transforming growth factor beta binding protein 1 | 2.26 | 2.23 |
| 81 | LOC644192 | uncharacterized LOC644192 | 2.24 | 2.10 |
| 82 | ZYG11B | zyg-11 homolog B (C. elegans) | 2.24 | 2.15 |
| 83 | RPL38 | ribosomal protein L38 | 2.24 | 2.35 |
| 84 | FAM98A | family with sequence similarity 98, member A | 2.23 | 2.06 |
| 85 | SRGAP2P1 | SLIT-ROBO Rho GTPase activating protein 2 pseudogene 1 | 2.22 | 2.15 |
| 86 | TFRC | transferrin receptor (p90, CD71) | 2.21 | 2.15 |
| 87 | RDH11 | retinol dehydrogenase 11 (all-trans/9-cis/11-cis) | 2.21 | 2.37 |
| 88 | ANKRD10 | ankyrin repeat domain 10 | 2.20 | 2.49 |
| 89 | NAPEPLD | N-acyl phosphatidylethanolamine phospholipase D | 2.19 | 2.11 |
| 90 | DENND5B | DENN/MADD domain containing 5B | 2.19 | 2.22 |
| 91 | NUPL1 | nucleoporin like 1 | 2.18 | 2.03 |
| 92 | ZNF667 | zinc finger protein 667 | 2.18 | 2.29 |
| 93 | NUDT12 | nudix (nucleoside diphosphate linked moiety X)-type motif 12 | 2.17 | 2.26 |
| 94 | FCF1 | FCF1 small subunit (SSU) processome component homolog | 2.16 | 2.53 |
| 95 | CRIM1 | cysteine rich transmembrane BMP regulator 1 (chordin-like) | 2.15 | 2.05 |
| 96 | ZNF566 | zinc finger protein 566 | 2.14 | 2.24 |
| 97 | RHEB | Ras homolog enriched in brain | 2.12 | 2.22 |
| 98 | C15orf57 | chromosome 15 open reading frame 57 | 2.06 | 2.16 |
| 99 | EIF3F | eukaryotic translation initiation factor 3, subunit F | 2.04 | 2.09 |
| 100 | PALMD | palmdelphin | 2.04 | 2.07 |
| 101 | SMC4 | structural maintenance of chromosomes 4 | 2.04 | 2.04 |
| 102 | FGFR2 | fibroblast growth factor receptor 2 | 2.03 | 2.14 |
| 103 | BCOR | BCL6 corepressor | 2.03 | 2.07 |
| 104 | MTPAP | mitochondrial poly(A) polymerase | 2.03 | 2.01 |
| 105 | STRN3 | striatin, calmodulin binding protein 3 | 2.03 | 2.05 |
| 106 | CCDC76 | coiled-coil domain containing 76 | 2.03 | 2.16 |
| 107 | ZNF678 | zinc finger protein 678 | 2.02 | 2.09 |
| 108 | FBXL20 | F-box and leucine-rich repeat protein 20 | 2.02 | 2.21 |
| 109 | NUP43 | nucleoporin 43kDa | 2.02 | 2.24 |
| 110 | PCLO | piccolo (presynaptic cytomatrix protein) | 2.02 | 2.07 |
| 111 | SSU72 | SSU72 RNA polymerase II CTD phosphatase homolog (S. cerevisiae) | 2.01 | 2.07 |

| **DOWN-REGULATED TRANSCRIPTS** | | | **Fold Change** | |
| --- | --- | --- | --- | --- |
| **No** | **Symbol** | **Gene name** | ***Sost^KO^*** | **DKK1** |
| 1 | PRKAB1 | protein kinase, AMP-activated, beta 1 non-catalytic subunit | 2.00 | 2.16 |
| 2 | HIVEP1 | human immunodeficiency virus type I enhancer binding protein 1 | 2.15 | 2.05 |
| 3 | PPP1R15A | protein phosphatase 1, regulatory subunit 15A | 2.17 | 2.04 |
| 4 | RHOB | ras homolog family member B | 2.22 | 2.14 |
| 5 | MGAT4B | mannosyl (α-1,3-)-glycoprotein beta-1,4-N-acetylglucosaminyltransferase, B | 2.31 | 2.35 |
