# Supplementary material for: SOST Inhibits Prostate Cancer Invasion
Source: PLoS One. 2015 Nov 6;10(11):e0142058. doi: 10.1371/journal.pone.0142058 (PMC4636315; doi:10.1371/journal.pone.0142058)
Supplement: S2 Table — Transcripts that are significantly changed in PC3 cells treated with rhSOST, but not significantly changed in PC3 cells co-cultured with Sost KO osteoblasts and DKK1. (DOCX) [file pone.0142058.s003.docx]

**S2 Table. Low invasive Genes**. Transcripts that are significantly changed in PC3 cells treated with rhSOST, but not significantly changed in PC3 cells co-cultured with *Sost^KO^* osteoblasts and DKK1

| **UP-REGULATED TRANSCRIPTS** | | | **Fold Change** |
| --- | --- | --- | --- |
| **No** | **Symbol** | **Gene Name** | **rhSOST** |
| 1 | MCTP1 | multiple C2 domains, transmembrane 1 | 2.36 |
| 2 | RPS24 | ribosomal protein S24 | 2.31 |
| 3 | CIRBP | cold inducible RNA binding protein | 2.30 |
| 4 | IL33 | interleukin 33 | 2.24 |
| 5 | NRK | Nik related kinase | 2.23 |
| 6 | PHACTR3 | phosphatase and actin regulator 3 | 2.16 |
| 7 | MGC16121 | uncharacterized protein MGC16121 | 2.14 |
| 8 | RBM15 | RNA binding motif protein 15 | 2.10 |
| 9 | SMAD4 | SMAD family member 4 | 2.08 |
| 10 | BCL6 | B-cell CLL/lymphoma 6 | 2.07 |
| 11 | ZNF182 | zinc finger protein 182 | 2.04 |
| 12 | MAP4K4 | mitogen-activated protein kinase kinase kinase kinase 4 | 2.03 |
| 13 | ATP5C1 | ATP synthase, H+ transporting, mitochondrial F1 complex, gamma polypeptide 1 | 2.03 |
| 14 | RAB3B | RAB3B, member RAS oncogene family | 2.02 |
| 15 | COL8A1 | collagen, type VIII, alpha 1 | 2.01 |

| **DOWN-REGULATED TRANSCRIPTS** | | | **Fold Change** |
| --- | --- | --- | --- |
| **No** | **Symbol** | **Gene name** | **rhSOST** |
| 1 | IWS1 | IWS1 homolog (S. cerevisiae) | 2.01 |
| 2 | IGF2BP3 | insulin-like growth factor 2 mRNA binding protein 3 | 2.02 |
| 3 | TRIM4 | tripartite motif containing 4 | 2.05 |
| 4 | CDCA2 | cell division cycle associated 2 | 2.07 |
| 5 | RIF1 | RAP1 interacting factor homolog (yeast) | 2.08 |
| 6 | KLF5 | Kruppel-like factor 5 (intestinal) | 2.09 |
| 7 | WAC | WW domain containing adaptor with coiled-coil | 2.09 |
| 8 | EHBP1 | EH domain binding protein 1 | 2.10 |
| 9 | ANKRD36B | ankyrin repeat domain 36B | 2.12 |
| 10 | PTPLB | protein tyrosine phosphatase-like (proline instead of catalytic arginine), member b | 2.13 |
| 11 | GBP1 | guanylate binding protein 1, interferon-inducible | 2.14 |
| 12 | SAMHD1 | SAM domain and HD domain 1 | 2.16 |
| 13 | CCDC13 | coiled-coil domain containing 13 | 2.19 |
| 14 | EGR1 | early growth response 1 | 2.19 |
| 15 | RASSF8 | Ras association (RalGDS/AF-6) domain family (N-terminal) member 8 | 2.19 |
| 16 | FERMT2 | fermitin family member 2 | 2.20 |
| 17 | NRIP1 | nuclear receptor interacting protein 1 | 2.21 |
| 18 | MOB1A | MOB kinase activator 1A | 2.22 |
| 19 | SETD2 | SET domain containing 2 | 2.22 |
| 20 | PPFIBP1 | PTPRF interacting protein, binding protein 1 (liprin beta 1) | 2.26 |
| 21 | USP10 | ubiquitin specific peptidase 10 | 2.26 |
| 22 | STYX | serine/threonine/tyrosine interacting protein | 2.27 |
| 23 | SPIN1 | spindlin 1 | 2.28 |
| 24 | USP2 | ubiquitin specific peptidase 2 | 2.28 |
| 25 | DDX17 | DEAD (Asp-Glu-Ala-Asp) box helicase 17 | 2.29 |
| 26 | ZNF641 | zinc finger protein 641 | 2.32 |
| 27 | USP16 | ubiquitin specific peptidase 16 | 2.34 |
| 28 | NFKBIA | nuclear factor of kappa light polypeptide gene enhancer in B-cells inhibitor, alpha | 2.36 |
| 29 | KLF6 | Kruppel-like factor 6 | 2.40 |
| 30 | TGFB2 | transforming growth factor, beta 2 | 2.40 |
| 31 | EVI5 | ecotropic viral integration site 5 | 2.40 |
| 32 | HECTD1 | HECT domain containing E3 ubiquitin protein ligase 1 | 2.45 |
| 33 | LIMA1 | LIM domain and actin binding 1 | 2.46 |
| 34 | SLC39A6 | solute carrier family 39 (zinc transporter), member 6 | 2.46 |
| 35 | CCDC88A | coiled-coil domain containing 88A | 2.50 |
| 36 | CXCL5 | chemokine (C-X-C motif) ligand 5 | 2.53 |
| 37 | FXR1 | fragile X mental retardation, autosomal homolog 1 | 2.54 |
| 38 | THRAP3 | thyroid hormone receptor associated protein 3 | 2.72 |
| 39 | ESCO2 | establishment of cohesion 1 homolog 2 (S. cerevisiae) | 2.81 |
| 40 | UGCG | UDP-glucose ceramide glucosyltransferase | 2.83 |
| 41 | LTN1 | listerin E3 ubiquitin protein ligase 1 | 2.92 |
| 42 | C10orf118 | chromosome 10 open reading frame 118 | 3.18 |
| 43 | FAM111B | family with sequence similarity 111, member B | 3.22 |
| 44 | OSBPL8 | oxysterol binding protein-like 8 | 3.50 |
| 45 | DHX9 | DEAH (Asp-Glu-Ala-His) box polypeptide 9 | 3.57 |
| 46 | PLEKHA2 | pleckstrin homology domain containing, family A member 2 | 3.65 |
| 47 | ESCO1 | establishment of cohesion 1 homolog 1 (S. cerevisiae) | 3.88 |
| 48 | ZFR | zinc finger RNA binding protein | 4.27 |
| 49 | MAP4K5 | mitogen-activated protein kinase kinase kinase kinase 5 | 4.85 |
| 50 | CDC27 | cell division cycle 27 homolog (S. cerevisiae) | 5.30 |
